# Supplementary material for: Drug treatment efficiency depends on the initial state of activation in nonlinear pathways
Source: Sci Rep. 2018 Aug 21;8:12495. doi: 10.1038/s41598-018-30913-9 (PMC6104077; doi:10.1038/s41598-018-30913-9)
Supplement: Supplementary file 1 — Supplementary Information [file 41598_2018_30913_MOESM1_ESM.pdf]

Supplementary Material: Drug treatment efficiency depends on the initial state of activation in nonlinear pathways

Victoria Doldán-Martelli, David G. Míguez*

July 16, 2018

High-throughput *in silico* screening of three-node networks. All programs and scripts have been developed in-house using Matlab© (The Mathworks©, Natick, MA). Code available as Supp. Material.

This computational high-throughput screening strategy is inspired on previous studies focused on network topologies that induce adaptation [1], bistability and ultrasensitivity [2]. The network is composed of three interacting nodes: an input node, which receives a constant stimulus, a target node that is affected by the presence of the inhibitor, and the output node, which serves as a readout of the network activity. We use a Michaelis-Menten type of interaction kinetics [3] between these three nodes that can result in reversible activation or de-activation of each other. We also allow direct positive and negative feedback in input and output nodes, and indirect feedback and feed-forward loops between all nodes. The set of three differential equations is therefore simplified as the following expression:

$$\frac{\partial X_j}{\partial t} = \sum_{i=1}^{9} (\delta_{(I_{i,j})(1)} \frac{(1 - X_j) \cdot X_i \cdot k_{i,j}}{K_{i,j} + 1 - X_j} - \delta_{(I_{i,j})(-1)} \frac{X_i \cdot X_j \cdot k_{i,j}}{K_{i,j} + X_j}) \tag{1}$$

As explained in the main text, X_j is the state vector ($j = 1, 2, 3$), where X_1 is the concentration of active input, X_2 is the target and X_3 is the output. We use normalized values for the total concentration thus, $1 - X_1$ is the concentration of the inactive form of the input node, $1 - X_2$ for the target and $1 - X_3$ for the output. The set of interactions for each topology are set in the interaction matrix, I, where a given component $I_{i,j}$ of the matrix is zero if X_i does not affect X_j, 1 if the X_i activates X_j and -1 if X_i deactivates X_j. The matrix takes the following explicit form:

$$I = \begin{pmatrix} I_{1,1} & I_{1,2} & I_{1,3} \\ I_{2,1} & 0 & I_{2,3} \\ I_{3,1} & I_{3,2} & I_{3,3} \\ I_{4,1} & 0 & 0 \\ 0 & I_{5,2} & 0 \\ 0 & 0 & I_{6,3} \\ I_{7,1} & 0 & 0 \\ 0 & I_{8,2} & 0 \\ 0 & 0 & I_{9,3} \end{pmatrix}$$

The first three rows define the interactions between input, target and output. The next rows define the effect of background enzymes that provide a constant stimulus that balances the activation or deactivation of X_1, X_2 and X_3 when they do not receive an activating or deactivation interaction, similarly to the approach used in Ref. [1]. This way, when a given node X_j does not receive a deactivating interaction from either itself or the other nodes, the corresponding background enzyme X_i is automatically added to compensate this, (otherwise the node will be always in its active form $X_j = 1$). The same occurs when a node does not receive an activating interaction (the corresponding deactivating background reaction is automatically set). For instance, if the target X_2 is not being activated by X_1 or X_3, the corresponding background activating enzyme (X_5 in this particular case) is used to activate the node (therefore $I_{5,2} = 1$). If, on the other hand, the output node X_3 does not receive a deactivating interaction, the corresponding deactivating enzyme (X_9)

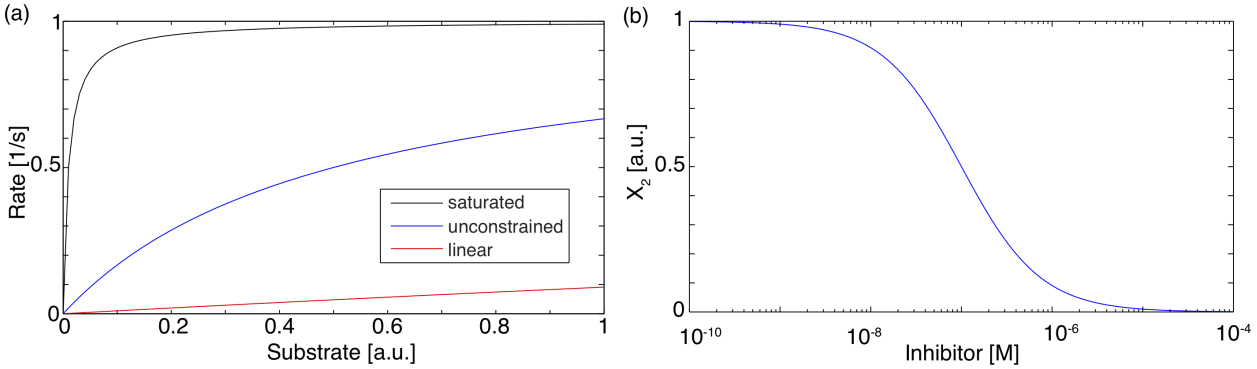

Supplementary Figure 1. (a) Profile of the rate of Michaelis-Menten kinetics for conditions of linear ($K_{i,j} >> 1$ [M]), unconstrained ($0.1 < K_{i,j} < 1$ [M]) and saturated ($K_{i,j} < 0.1$ [M]) regimes. (b) Concentration of active target X_2 depending on the concentration of inhibitor. $k_+ = 10^7$ [$s^{-1}M^{-1}$], $k_- = 1$ [s^{-1}].

is automatically introduced in the topology to compensate all activating interaction that act on X_3 (in this case $I_{9,3} = -1$).

To automatically set the summands that are active in each equation, we use Kronecker delta functions $\delta_{(I_{i,j})(1)}$ and $\delta_{(I_{i,j})(-1)}$, that are nonzero when the value $I_{i,j}$ is 1 or -1, respectively. This way, if X_i activates X_j the left part of the subtraction is nonzero, while if X_i deactivates X_j, only the right summand is nonzero.

Parameters $k_{i,j}$ and $K_{i,j}$ are the components of the k and K matrices, corresponding to the kinetic rate and Michaelis-Menten constants for the interaction of X_i on X_j. For each given topology, we generate 10000 different k and K matrices where each component of the matrix is obtained from a uniform distribution in logarithmic scale between values 0.1 and 10 for $k_{i,j}$, and between 10^{-3} and 10^2 for the Michaelis-Menten constant ($K_{i,j}$). Taking into account that the concentration of all substrates is normalized to 1, the range for the Michaelis-Menten constant allows us to sample the regime of saturated $K_{i,j} < 0.1$, unconstrained $0.1 < K_{i,j} < 1$ and linear $K_{i,j} >> 1$ regimes for the Michaelis-Menten dynamics. The kinetics for three values illustrating these regimes are plotted in Supp. Fig. 1a. These regimes are illustrated in the box plots in Figures 5, 6 and Supp. Fig. 7 as blue, green and red background colors, respectively.

In addition, a constant stimulus in the input node maintains a constant stimulation at intermediate levels of the input node. The dynamics of this stimulus is set as:

$$X_1(t) = X_1^{max}(1 - e^{-t \cdot k_{stimulus}}) \tag{2}$$

Therefore, the following term is added in its differential form to the node X_1 in all simulations:

$$dX_1/dt = (X_1^{max} - X_1) \cdot k_{stimulus} \tag{3}$$

For our simulations, the value of $k_{stimulus}$ is fixed at 0.1 [s^{-1}]. For simplicity, the concentration of all background enzymes is fixed at 0.5 (i.e., $X_{4...9} = 0.5$ in the state vector X). The maximum value of X_1 at $t \to \infty$ is set at $X_1^{max} = 0.5$.

Modeling Small Molecule Inhibition. Fundamentally, a chemical inhibitor acts by reversibly binding to its target to reduce or block its enzymatic activity [4], following the scheme:

$$[X_2] + [inh] \underset{\mathrm{k}_-}{\overset{\mathrm{k}_+}{\rightleftarrows}} [X_2 - inh] \tag{4}$$

that takes into account the reversible binding of inhibitor inh and its target X_2, to form a complex $X_2 - inh$. Ideally, inhibitors should have high rates of binding (k_+) and slow rates of unbinding (k_-) to

maximize residence time with the target enzyme [5]. If these values are around 10^6 $s^{-1}M^{-1}$ for k_+ and 10^{-2} s^{-1} for k_-, as reported form some small molecules inhibitors in the literature [6], equilibrium is reached within a few seconds. This equilibrium concentrations is:

$$[X_2 - inh]_{eq} = \frac{k_+}{k_-}[X_2]_{eq} \cdot [inh]_{eq} = K_a \cdot [X_2]_{eq} \cdot [inh]_{eq} \tag{5}$$

where $K_a = k_+/k_-$ is the association constant of the interaction. Typically, the amount of molecules present for the inhibitor is several orders of magnitude higher than the target (experimental working inhibitor concentrations are often in the range of 10^{-9}... 10^{-6} M), so $[X_2 - inh]_{eq} << [inh]_{eq}$ [7] (our estimation for a highly expressed protein and our lowest inhibition concentration is that the amount of molecules of inhibitor in solution is at least two orders of magnitude higher than the amount of molecules of protein). In these conditions, we can safely assume that the concentration of inhibitor remains constant, so $[inh]_{eq} \approx [inh]_0$ (initial concentration of inhibitor). Also, taking into account the conservation of total amount of active target molecule $[X_2]_T = [X_2 - inh]_{eq} + [X_2]_{eq}$, we can rewrite Eq. 5 as:

$$[X_2]_T - [X_2]_{eq} = K_a \cdot [X_2]_{eq} \cdot [inh]_0 \tag{6}$$

that rearranging terms becomes,

$$[X_2]_{eq} = \frac{[X_2]_T}{1 + K_a \cdot [inh]_0} \tag{7}$$

This equation expresses the equilibrium concentration of active target X_2 in terms of the total amount of active target $[X_2]_T$ and the initial concentration of inhibitor $[inh]_0$. The typical shape of this curve is shown in Supp. Fig. 1b.

We assume the general case of reversible non-competitive inhibition, i.e, the inhibitor is a small compound that binds to the active site of the target reducing its activity, without affecting the binding to its substrate. This way, the inhibitor does not affect the K_M of the interaction between enzyme and substrate. Under these conditions, when $i = 2$ in Eq. 1, X_2 can be substituted by $[X_2]_{eq}$ calculated via Eq. 8. This is equivalent to substituting the kinetic constant $k_{i,j}$ when $i = 2$ by an effective value $k_{i,j}/(1 + K_a \cdot [inh]_0)$. In our simulations, the value of K_a is fixed at 10^7 $1/M$, so the inhibitor is active in the range of μM to nM concentrations, as most experimental small molecule inhibitors (see Supp. Fig. 1b.).

This approximation allows us to automatically perform the screening of all possible topologies using the same set of equations independently of the interactions where X_2 is involved. To test the validity of this equilibrium approximation, we performed numerical simulations where we compared the dynamics of the activation of the output X_3 using mass action (Supp. Fig. 2a) and the equilibrium approximation (Supp. Fig. 2b). The output is plotted in Supp. Fig. 2c-d, for different kinetics of binding k_+ and unbinding k_- but maintaining the same value of $K_a = k_+/k_-$.

When values of k_+ around 10^5 $s^{-1}M^{-1}$ are used, the amount of active target X_3 using mass action (yellow curve) and equilibrium approach (black dotted curve) do not match even after 60 seconds of the simulation. On the other hand, when we use values of k_+ closer to 10^6 $s^{-1}M^{-1}$, comparable to several small molecule inhibitors found in the literature [6], the equilibrium approximation is equivalent to the mass action dynamics after 15 seconds.

Unfortunately, the equilibrium approximation cannot be used in conditions where the target node is activating or deactivating itself. Therefore, in these networks, the effect of the inhibitor cannot be simplified as an effective concentration of active X_2 at equilibrium, and full mass action has to be simulated explicitly as a sequestering interaction. To study these types of topologies, a different set of equations has to be written for each particular network topology, and the simulation and analysis can not be automated using the same script for all topologies. Because of this, we have to exclude from our analysis the possibility of positive or negative autoregulation in X_2 (i.e, $I_{2,2} = 0$ in all interaction matrices), and therefore the number of possible network topologies that our automated screening can test is reduced (see next section).

Calculation of all possible network topologies. The first three rows of the interaction matrix I are:

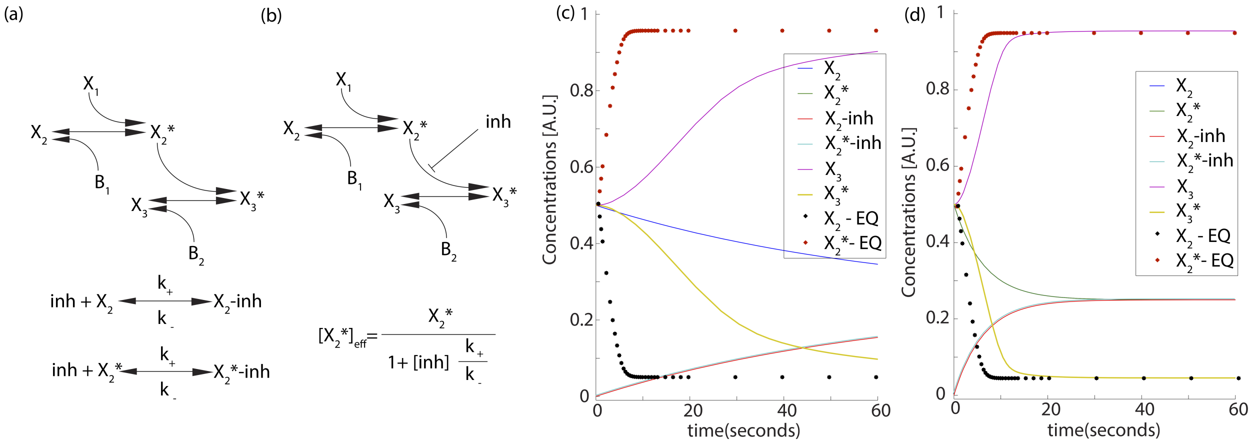

Supplementary Figure 2: Comparison of mass-action dynamics and quasi-steady state approximation for inhibitor concentration. (a-b) Scheme of interaction for the (a) mass action simulation and (b) quasi-steady state approximation. (c-d) Simulations for (c) slow and (d) fast dynamics of inhibitor binding. Solid curves correspond to mass action simulations, dotted curves correspond to the steady state assumption. Kinetic constants for slow binding: $k_+ = 8 \cdot 10^4\ s^{-1}M^{-1}$, $k_- = 8 \cdot 10^{-2}\ s^{-1}$. Kinetic constants for values of binding and unbinding similar to experimental data [6]: $k_+ = 8 \cdot 10^5\ s^{-1}M^{-1}$, $k_- = 8 \cdot 10^{-3}$ s^{-1}.

$$I = \begin{pmatrix} I_{1,1} & I_{1,2} & I_{1,3} \\ I_{2,1} & I_{2,2} & I_{2,3} \\ I_{3,1} & I_{3,2} & I_{3,3} \end{pmatrix}$$

Where each component $I_{i,j}$ can be 1, -1 or 0, depending on wether component X_i activates, deactivates or does not affect X_j, respectively. $I_{i,j} = 0$ if X_i does not act on X_j. To calculate all possible topologies, we consider all 3 potential types of interaction between the three nodes. This gives us a total number of possible networks of $N = 3^{3\times3} = 19683$.

Based on the previous section, all network topologies that contain positive or negative autoregulation of the target node are not considered in our analysis. These topologies correspond to the interaction matrices with the following form:

$$I_{i,j} = \begin{pmatrix} I_{1,1} & I_{1,2} & I_{1,3} \\ I_{2,1} & 1 & I_{2,3} \\ I_{3,1} & I_{3,2} & I_{3,3} \end{pmatrix} \text{ or } I_{i,j} = \begin{pmatrix} I_{1,1} & I_{1,2} & I_{1,3} \\ I_{2,1} & -1 & I_{2,3} \\ I_{3,1} & I_{3,2} & I_{3,3} \end{pmatrix}$$

Therefore, we only take into account the networks where the interaction $I_{2,2} = 0$, resulting in $N = 3^{(3\times3)-1} =$ 6561 networks.

Next, since we are interested in the effect of inhibition, we do not consider the topologies where the target node X_2 does not act on the other two nodes, i.e., all network topologies that take the form:

$$I_{i,j} = \begin{pmatrix} I_{1,1} & I_{1,2} & I_{1,3} \\ 0 & 0 & 0 \\ I_{3,1} & I_{3,2} & I_{3,3} \end{pmatrix}$$

That constitute a total of $N = 3^{(3\times3)-3} = 729$ network topologies. Finally, we eliminate from our analysis all topologies where the output does not receive an interaction from the other two nodes, i.e., all interaction matrices that take the form:

$$I_{i,j} = \begin{pmatrix} I_{1,1} & I_{1,2} & 0 \\ I_{2,1} & 0 & 0 \\ I_{3,1} & I_{3,2} & I_{3,3} \end{pmatrix}$$

Which constitute another $N = 3^{(3\times3)-3} = 729$ network topologies. Considering this, the total number

of possible network topologies evaluated in our screening is N=5103.

Detailed description of the workflow

1. A given network topology is set, defined by its interaction matrix $I_{i,j}$.
2. The matrices of kinetic ($k_{i,j}$) and Michaelis-Menten constants $K_{i,j}$ are populated with random values obtained from a uniform distribution in logarithmic scale.
3. The initial conditions for X_1, X_2 and X_3 are set from a uniform distribution between 0 and 1.
4. The system of three equations for X_1, X_2 and X_3 is numerically solved, applying two different constant concentrations of inhibitor ($[inh]_{low} = 0$ nM and $[inh]_{high} = 10^3$ nM).
5. The two stable steady states obtained for low $[X_1^{ss}, X_2^{ss}, X_3^{ss}]_{low}$ and high $[X_1^{ss}, X_2^{ss}, X_3^{ss}]_{high}$ inhibitor concentrations are now used as initial conditions IC_{low} and IC_{high} for new numerical simulations, where different constant concentrations of inhibitor (range for $[inh]$= 0-10^3 nM) are applied.
6. The new steady state values of the target, $[X_3^{ss}]_{low}$ and $[X_3^{ss}]_{high}$ obtained for each concentration of inhibitor applied are plotted against the concentration of inhibitor, to generate two dose-response curves, DS_{low} and DS_{high},where the subindexes *low* and *high* refer to the initial conditions used in the simulation.
7. The shape and other relevant characteristics of the two dose-response curves are analyzed, compared and classified using a semi-automated script to determine the network topologies that can induce dependence on the initial conditions

Steps 2 to 7 are repeated 10000 times for each topology, to sample the parameter space and determine regions where the network induces dependence of initial conditions. This sampling of the parameter space also allows us to test the cases where the dose-response changes even in conditions where bistability occurs at low or high inhibitor. Steps 1 to 7, are repeated 5103 times to sample all relevant topologies for our study (see below).

For instance, let's assume one of the simplest possible topologies where X_1 activates X_2, while X_2 inactivates X_3. The first three rows of the interaction matrix I for this particular topology take the form:

$$I_{i,j} = \begin{pmatrix} 0 & 1 & 0 \\ 0 & 0 & -1 \\ 0 & 0 & 0 \end{pmatrix}$$

Since the input and target nodes do not receive an inactivating interaction, the components of the interaction matrix for the corresponding inactivating background enzymes are set ($I_{7,1}$ and $X_{8,2}$). In addition, since the output node does not receive an activating interaction, the components of the interaction matrix for the corresponding activating background enzymes are set ($I_{6,3}$). The input node always receives an activating interaction (calculated in Eq. 3), that mimics a constant upstream activation of the pathway, therefore it does not require an activating background. This way, the interaction matrix takes the form:

$$I = \begin{pmatrix} 0 & 1 & 0 \\ 0 & 0 & -1 \\ 0 & 0 & 0 \\ 0 & 0 & 0 \\ 0 & 0 & 0 \\ 0 & 0 & 1 \\ -1 & 0 & 0 \\ 0 & -1 & 0 \\ 0 & 0 & 0 \end{pmatrix}$$

So, the second and third columns of the interaction matrix I have at least a 1 and a -1, meaning that they receive at least an activating and a deactivating interaction (the input already receives the constant activation of the stimulus).

Next, the script generates 10000 different matrices for the kinetic rate k and Michaelis-Menten constants obtained from a uniform distribution in logarithmic scale. Two examples of each of the matrices for this particular topology are:

$$k = \begin{pmatrix} 0 & 0.3182 & 0 \\ 0 & 0 & 1.0418 \\ 0 & 0 & 0 \\ 0 & 0 & 0 \\ 0 & 0 & 0 \\ 0 & 0 & 0.57 \\ 8.37 & 0 & 0 \\ 0 & 4.87 & 0 \\ 0 & 0 & 0 \end{pmatrix}$$

$$K = \begin{pmatrix} 0 & 5.70 & 0 \\ 0 & 0 & 2.06 \\ 0 & 0 & 0 \\ 0 & 0 & 0 \\ 0 & 0 & 0 \\ 0 & 0 & 0.057 \\ 0.0067 & 0 & 0 \\ 0 & 0.1430 & 0 \\ 0 & 0 & 0 \end{pmatrix}$$

Once the parameters are set, the script performs the numerical simulations for the dynamics of input, target and output nodes. These three differential equations are shown below:

$$\frac{\partial X_1}{\partial t} = \sum_{i=1}^{9}(\delta_{(I_{i,1})(1)}\frac{(1-X_1)\cdot X_i \cdot k_{i,1}}{K_{i,1}+1-X_1} - \delta_{(I_{i,1})(-1)}\frac{X_i \cdot X_1 \cdot k_{i,1}}{K_{i,1}+X_1}) + (0.5-X_1)\cdot k_{stimulus} \quad (8)$$

$$\frac{\partial X_2}{\partial t} = \sum_{i=1}^{9}(\delta_{(I_{i,2})(1)}\frac{(1-X_2)\cdot X_i \cdot k_{i,2}}{K_{i,2}+1-X_2} - \delta_{(I_{i,2})(-1)}\frac{X_i \cdot X_2 \cdot k_{i,2}}{K_{i,2}+X_2}) \quad (9)$$

$$\frac{\partial X_3}{\partial t} = \sum_{i=1}^{9}(\delta_{(I_{i,3})(1)}\frac{(1-X_3)\cdot X_i \cdot k_{i,3}}{K_{i,3}+1-X_3} - \delta_{(I_{i,3})(-1)}\frac{X_i \cdot X_3 \cdot k_{i,3}}{K_{i,3}+X_3}) \quad (10)$$

where the last term in Eq. 8 correspond to the constant stimulation of the input node. Then, the Kronecker delta functions set all the correct activating $\delta_{(I_{i,j})(1)}$ and deactivating interactions $\delta_{(I_{i,j})(-1)}$ in their corresponding summand of each equation. For this particular example, equations 8-10 take the explicit form:

$$\frac{\partial X_1}{\partial t} = (0.5-X_1)\cdot k_{stimulus} - \frac{X_7 \cdot X_1 \cdot k_{7,1}}{K_{7,1}+X_1} \quad (11)$$

$$\frac{\partial X_2}{\partial t} = \frac{(1-X_2)\cdot X_1 \cdot k_{1,2}}{K_{1,2}+1-X_2} - \frac{X_8 \cdot X_2 \cdot k_{8,2}}{K_{8,2}+X_2} \quad (12)$$

$$\frac{\partial X_3}{\partial t} = \frac{(1-X_3)\cdot X_6 \cdot k_{6,3}}{K_{6,3}+1-X_3} - \frac{X_2 \cdot X_3 \cdot k_{2,3}}{K_{2,3}+X_3} \quad (13)$$

Since the concentration of the background enzymes does not change over time, the concentration values of

$X_{4...9} = 0.5$ can be substituted in the equations:

$$\frac{\partial X_1}{\partial t} = (0.5 - X_1) \cdot k_{stimulus} - \frac{0.5 \cdot X_1 \cdot k_{7,1}}{K_{7,1} + X_1} \quad (14)$$

$$\frac{\partial X_2}{\partial t} = \frac{(1 - X_2) \cdot X_1 \cdot k_{1,2}}{K_{1,2} + 1 - X_2} - \frac{0.5 \cdot X_2 \cdot k_{8,2}}{K_{8,2} + X_2} \quad (15)$$

$$\frac{\partial X_3}{\partial t} = \frac{(1 - X_3) \cdot 0.5 \cdot k_{6,3}}{K_{6,3} + 1 - X_3} - \frac{X_2 \cdot X_3 \cdot k_{2,3}}{K_{2,3} + X_3} \quad (16)$$

We finally incorporate the effect of the inhibitor by substituting in equations 14-16 the value of X_2 by the expression in Eq. 7, whenever is acting as an activating or deactivating enzyme (i.e., not as a substrate of the other enzymes). In this particular example X_2 only affects the value of X_3 via equation 16, so the full set of equations takes the form:

$$\frac{\partial X_1}{\partial t} = (0.5 - X_1) \cdot k_{stimulus} - \frac{0.5 \cdot X_1 \cdot k_{7,1}}{K_{7,1} + X_1} \quad (17)$$

$$\frac{\partial X_2}{\partial t} = \frac{(1 - X_2) \cdot X_1 \cdot k_{1,2}}{K_{1,2} + 1 - X_2} - \frac{0.5 \cdot X_2 \cdot k_{8,2}}{K_{8,2} + X_2} \quad (18)$$

$$\frac{\partial X_3}{\partial t} = \frac{(1 - X_3) \cdot 0.5 \cdot k_{6,3}}{K_{6,3} + 1 - X_3} - \frac{\frac{X_2}{1+K_a \cdot inh} \cdot X_3 \cdot k_{2,3}}{K_{2,3} + X_3} \quad (19)$$

The numerical simulation of these equations is then performed for each of the 10000 k and K matrices, at two concentrations of inhibitor ($[inh]_{low} = 0$ nM and $[inh]_{high} = 10^3$ nM). These two sets of steady state values ($[X_1^{ss}, X_2^{ss}, X_3^{ss}]_{low}$ and $[X_1^{ss}, X_2^{ss}, X_3^{ss}]_{high}$) are then used as initial conditions for new numerical simulations where different constant concentrations of inhibitor are applied to generate the two dose-response curves corresponding to each initial condition, as explained in the main text.

Organization of all network topologies that show inverse bistability and inverse hysteresis.

The 712 topologies that showed inverse bistability are organized in a undirected graph or *complexity atlas*, similarly to Ref. [8]. Herein, the topologies are represented by nodes while the lines represent the similarity between topologies: Linked topologies (neighbors) present the same connectivity pattern except for one interaction, i.e., we can go from one to another neighbor by adding or removing a single interaction. The resulting connectivity matrix was reorganized by number of links and plotted using the *biograph* function included in the Bioinformatics Toolbox of Matlab (The Mathworks, Natick, MA). The 19 minimal motifs are represented as matrix plots, taking into account the three first rows of the interaction matrix for each of the topologies. This way, a positive interaction from species i to species j of the network (i.e.,"1" in the component $I_{i,j}$ of the interaction matrix) is represented as a white square; a black square corresponds to a negative interaction, ("-1" in the component $I_{i,j}$ of the interaction matrix) while a grey square means that there is no interaction ("0" in the component $I_{i,j}$ of the interaction matrix).

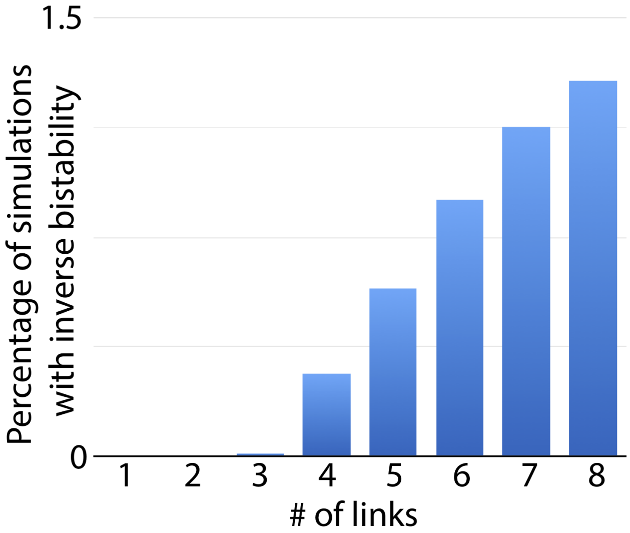

Supplementary Figure 3: The percentage of simulations with inverse bistability increases with the connectivity of the network.

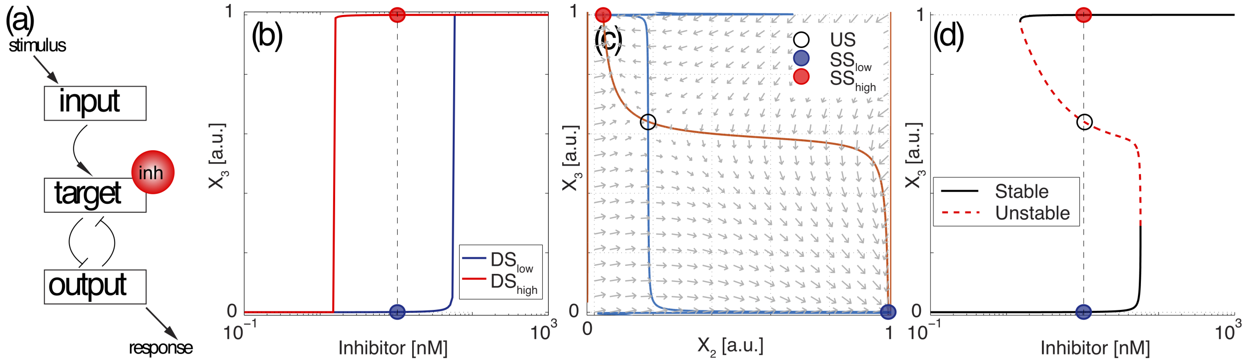

Supplementary Figure 4: Dependence on the initial conditions with the inhibitor acting as activator of the output node.. Panel (a) represents the network topology used in this simulation. Pointed arrows represent positive interactions (activation) and blunt arrows represents negative interactions (de-activation). Panel (b) represents the dose-response curves DS_{low} (blue) and DS_{high} red for initial conditions IC_{low} and IC_{high}, respectively. The rest of parameter values are the same between the two curves. Blue and red circles SS_{low} and SS_{high} show the steady state solutions for a given concentration of inhibitor. Panel (c) represents the phase plane with vector field and nullclines, representing the two steady states SS_{low} (blue) and SS_{high} (red) respectively. Panel (d) shows the bifurcation diagram of X_3. Black curves are the stable branches and the dash red curve is the unstable branch.

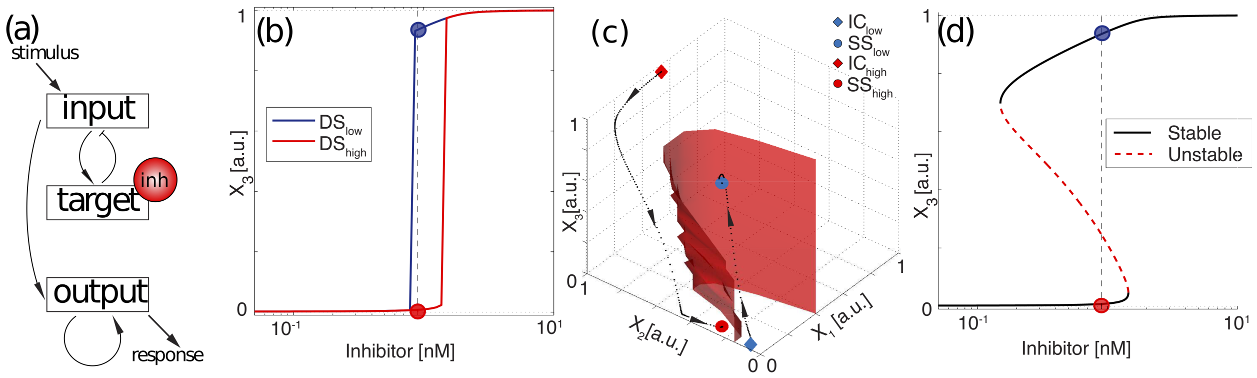

Supplementary Figure 5: Inverse bistability in conditions where the drug acts as an activator of the output node. Panel (a) represents an examples of network topology that show inverse bistability. Pointed arrows represent positive interactions (activation) and blunt arrows represents negative interactions (de-activation). Panel (b) represents the dose response curves DS_{low} (blue) and DS_{high} red for initial conditions IC_{low} and IC_{high}, respectively. The rest of parameter values are the same between the two curves. Blue and red circles SS_{low} and SS_{high} represent the steady state solutions for a given concentration of inhibitor. Panel (c) represents the three-dimensional phase plane, with the trajectories of each simulation starting from the two initial conditions, and the separatrix between the two basins of attraction. Panel (d) shows the bifurcation diagram of X_3. Black curves are the stable branches and the red dash curve is the unstable branch.

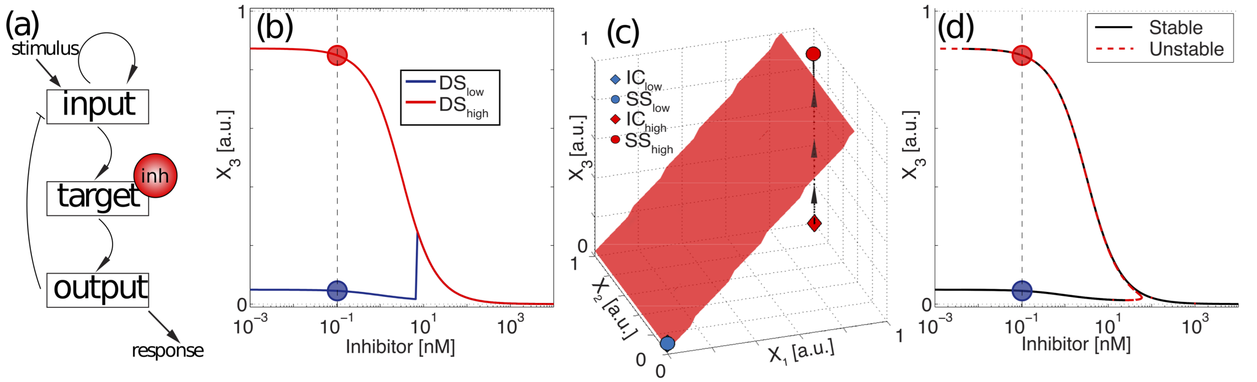

Supplementary Figure 6: The network architecture can induce Inverse Irreversible Hysteresis. (a) Example of a network architecture that induce inverse hysteresis but one of the dose-response curves becomes insensitive to treatment. Pointed arrows represent positive interactions (activation) and blunt arrows represents negative interactions (de-activation). (b) Dose-response curves DS_{low} (blue) and DS_{high} red for initial conditions IC_{low} and IC_{high}, respectively. The rest of parameter values are the same between the two curves. Blue and red circles SS_{low} and SS_{high} represent the steady state solutions for a given concentration of inhibitor. Panel (c) represents the three-dimensional phase plane, with the trajectories of each simulation starting from the two initial conditions, and the separatrix between the two basins of attraction. (d) Bifurcation diagram of X_3. Black curves are the stable branches and the dash red curve is the unstable branch.

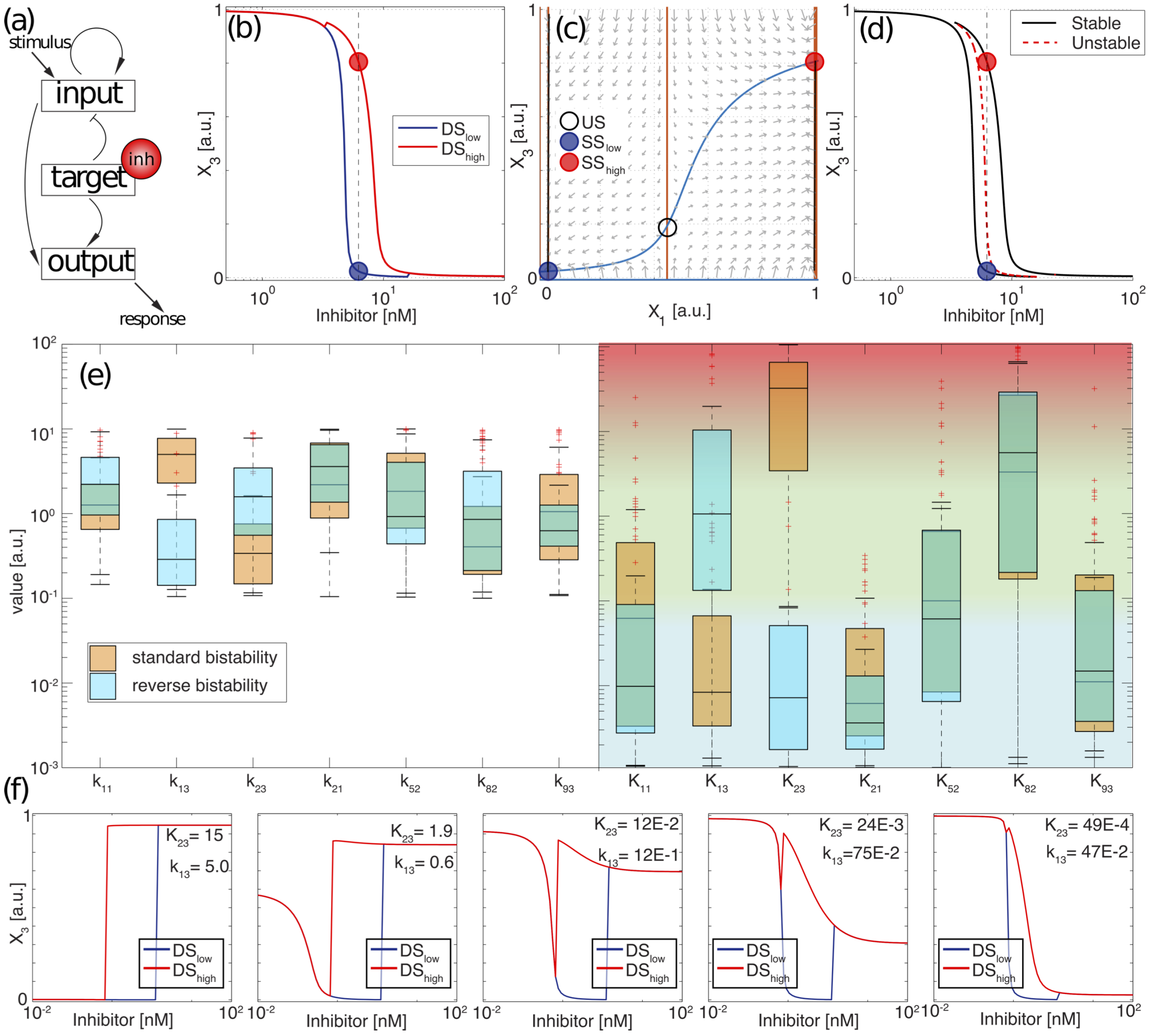

Supplementary Figure 7: The network architecture can induce Inverse Hysteresis. (a) Example of a network architecture that induce inverse hysteresis. Pointed arrows represent positive interactions (activation) and blunt arrows represents negative interactions (de-activation). (b) Dose-response curves DS_{low} (blue) and DS_{high} red for initial conditions IC_{low} and IC_{high}, respectively. The rest of parameter values are the same between the two curves. Blue and red circles SS_{low} and SS_{high} show the steady state solutions for a given concentration of inhibitor. (c) Phase plane with vector field and nullclines, representing the two steady states SS_{low} (blue) and SS_{high} (red) respectively. (d) Bifurcation diagram of X_3. Black curves are the stable branches and the red dash curve is the unstable branch. (e) Box plot for all parameter sets that show standard and inverse hysteresis. Blue, green and red background represents the saturated, unconstrained and linear regimes of the Michaelis-Menten kinetics, respectively. (f) Changes in the dose-response curve when two parameters are changed from standard to inverse hysteresis conditions.

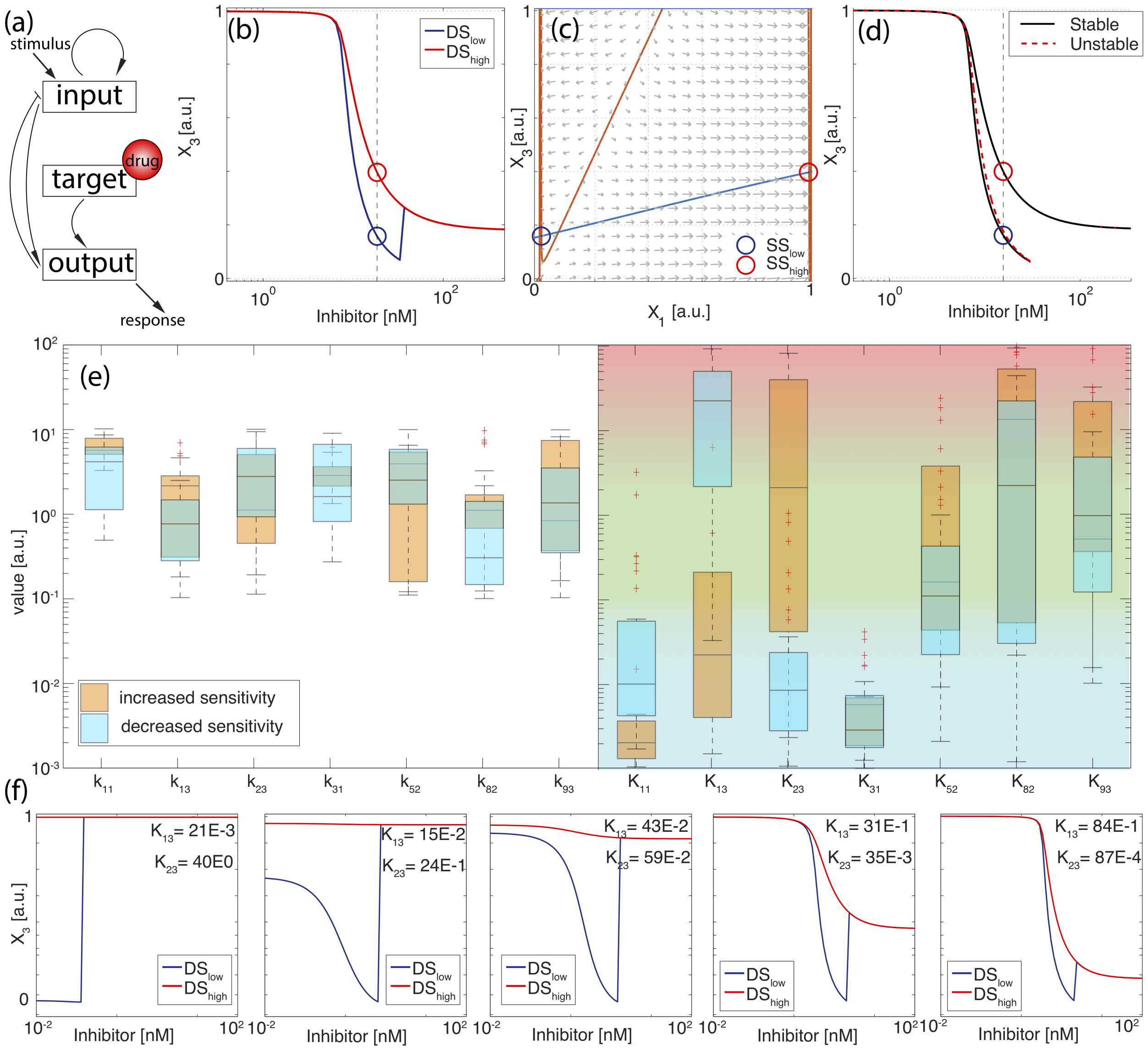

Supplementary Figure 8: The network architecture can induce inverse hysteresis. Here, the standard bistability is irreversible, i.e., X_3 is insensitive to changes in the inhibitor for one of the initial conditions. a) Example of a network architecture that induces inverse hysteresis. Pointed arrows represent positive interactions (activation) and blunt arrows represents negative interactions (de-activation). (b) Dose-response curves DS_{low} (blue) and DS_{high} red for initial conditions IC_{low} and IC_{high}, respectively. The rest of parameter values are the same between the two curves. Blue and red circles SS_{low} and SS_{high} represent the steady state solutions for a given concentration of inhibitor. For this inhibitor concentration, we plot the corresponding phase plane (c) with vector field and nullclines for X_3 (blue) and X_1 (orange), representing two stable steady states (SS_{low} in blue, and SS_{high} in red), and an unstable steady state US (empty black circle). (d) Bifurcation diagram of X_3. Black curves are the stable branches and the red dash curve is the unstable branch. (e) Box plot for all parameter sets that show standard irreversible bistability and inverse bistability, showing that parameters $K_{1,3}$ and $K_{2,3}$ do not overlap for both standard (orange) and inverse (blue) bistability. Blue, green and red background represents the saturated, unconstrained and linear regimes of the Michaelis-Menten kinetics, respectively. Activation of X_3 by X_1 changes from saturated to linear, affecting mainly the dose-response curves at high inhibitor concentrations. Activation of X_3 by X_2 becomes saturated, mainly affecting the regime at low inhibitor concentrations. Combination of these two effects, while maintaining a bistable regime at intermediate inhibitor concentrations, is able to transform an irreversible standard bistability to an inverse bistability. (f) Evolution of the dose-response curves when $K_{1,3}$ and $K_{2,3}$ are simultaneously changed from their average value at standard and inverse bistability (the rest of parameters are kept constant and correspond to the average of the means of the two distributions).

Video S1: Animation of dual response to inhibition in conditions of standard bistability. (a) Dose-response curves DS_{low} (blue) and DS_{high} red for initial conditions IC_{low} and IC_{high}, respectively. The rest of parameter values are the same between the two curves. (b) Phase plane with vector field and nullclines, representing the two steady states SS_{low} (blue) and SS_{high} (red) respectively. (c) Bifurcation diagram of X_3. Black curves are the stable branches and the red dash curve is the unstable branch.

Video S2: Animation of dual response to inhibition in conditions of inverse bistability. (a) Dose-response curves DS_{low} (blue) and DS_{high} red for initial conditions IC_{low} and IC_{high}, respectively. The rest of parameter values are the same between the two curves. (b) Three-dimensional phase plane, with the trajectories of each simulation starting from the two initial conditions. (c) Bifurcation diagram of X_3. Black curves are the stable branches and the red dash curve is the unstable branch.

Video S3: Animation of dual response to inhibition in conditions of irreversible inverse bistability. (a) Dose-response curves DS_{low} (blue) and DS_{high} red for initial conditions IC_{low} and IC_{high}, respectively. The rest of parameter values are the same between the two curves. (b) Phase plane with vector field and nullclines, representing the two steady states SS_{low} (blue) and SS_{high} (red) respectively. (c) Bifurcation diagram of X_3. Black curves are the stable branches and the red dash curve is the unstable branch.

Video S4: Animation of dual response to inhibition in conditions of inverse hysteresis. Please note that in this animation, unlike the rest of videos, the concentration of inhibitor is gradually increased and then decreased to illustrate the hysteresis loop. (a) Dose-response curves $DS_{increasing}$ (blue) and $DS_{decreasing}$ (red) for increasing and decreasing initial conditions, respectively. The rest of parameter values are the same in the two curves. For each inhibitor concentration (represented as a vertical dashed line in (a)) the phase plane is represented, with vector field and nullclines, showing the two stable steady states $SS_{increasing}$ (blue) and $SS_{decreasing}$ (red). (c) Bifurcation diagram of X_3. Black curves are the stable branches and the red dash curve is the unstable branch.

References

[1] Ma, W., Trusina, A., El-Samad, H., Lim, W. A. & Tang, C. Defining network topologies that can achieve biochemical adaptation. *Cell* **138**, 760–73 (2009).

[2] Shah, N. A. & Sarkar, C. A. Robust Network Topologies for Generating Switch-Like Cellular Responses. *PLoS Computational Biology* **7**, e1002085 (2011).

[3] Chen, W. W., Niepel, M. & Sorger, P. K. Classic and contemporary approaches to modeling biochemical reactions. *Genes & development* **24**, 1861–75 (2010).

[4] Vogel, R. M., Erez, A. & Altan-Bonnet, G. Dichotomy of cellular inhibition by small-molecule inhibitors revealed by single-cell analysis. *Nature communications* **7**, 12428 (2016).

[5] Shokat, K. M. *Methods in enzymology. Volume 584, Protein kinase inhibitors in research and medicine* (Elsevier, 2014), 1st edn.

[6] Thurmond, R. L., Wadsworth, S. A., Schafer, P. H., Zivin, R. A. & Siekierka, J. J. Kinetics of small molecule inhibitor binding to p38 kinase. *European journal of biochemistry* **268**, 5747–54 (2001).

[7] Doldán-Martelli, V., Guantes, R. & Míguez, D. G. A mathematical model for the rational design of chimeric ligands in selective drug therapies. *CPT: pharmacometrics & systems pharmacology* **2**, e26 (2013).

[8] Cotterell, J. & Sharpe, J. An atlas of gene regulatory networks reveals multiple three-gene mechanisms for interpreting morphogen gradients. *Molecular Systems Biology* **6**, 425 (2010).
