# Supplementary material for: Drug treatment efficiency depends on the initial state of activation in nonlinear pathways
Source: Sci Rep. 2018 Aug 21;8:12495. doi: 10.1038/s41598-018-30913-9 (PMC6104077; doi:10.1038/s41598-018-30913-9)
Supplement: Supplementary file 6 — Matlab scripts [file 41598_2018_30913_MOESM6_ESM.zip › Network_Matlab_scripts/dresfun_it.pdf]

```
%Calls latin.m
%calls rcalcfun_fixed.m

function Masterout=dresfun_it(Master,maxit) %dose response function
for it=1:maxit
    fprintf('Iteration=%i\n',it)
    % clearvars -except Master
    % clearvars -global -except Master
    global  kprev kinprev K Kin kbk Kbk bk cstim stim Lvec nod
M%latin_type
    % For constant stimuli towards A.
    stim=0.5; cstim=0.1;
    rvec=[];
    rvecpos=[];
    yc0=rand(1,nod); %Random initial values for 1st exposure.
    Matrix=Master{1,1}; Matrix(Matrix==2)=-1; M=Matrix;

    [kmat, Kmat]=latin(nod,1); %random screening of kinetic
parameters using latin hypercube.
    kprev=kmat{1,1}; kinprev=kmat{1,2}; kbk=kmat{1,3};
    K=Kmat{1,1}; Kin=Kmat{1,2}; Kbk=Kmat{1,3};

    bk=bg(nod,M);

    %ONLY ONE RUN:
    rerun=0;
    [rvec, rvecpos, rvecA, rvecposA,rvecB,rvecposB, high_yc,
sserror,warn,yc0_1,yc0_2]= rcalcfun_fixed(yc0);
    if sum(isnan(rvec))>0 || sum(isnan(rvecpos))>0 ||
length(rvec)<length(Lvec) || length(rvecpos)<length(Lvec)% NaNs or
incomplete?
        rerun=1; %rerun_marker=1
    end

    %% Storage of results in R:
    R.('initial_response')={rvec};
    R.('after_respose')={rvecpos};
    R.('K')=Kmat;
    R.('k')=kmat;
    R.('L')={Lvec};
    R.('datetime')= datestr(clock,20);
    R.('initial_conc')=yc0_1;
    R.('max_conc')=yc0_2;
    R.('high_yc')={high_yc};
    R.('rerun')=rerun;
    R.('sserror')={sserror};
    R.('warn')={warn};
    %% Storage of results in Master:
    if isempty(Master{1,2})== 1 %never added a value to that
topology.
        Master{1,2}=R;
    else
        fnames = fieldnames(R);
        for j = 1:length(fnames)
```

```
            try
                Master{1,2}.(fnames{j})= [Master{1,2}.(fnames{j});R.
(fnames{j})];
            catch
                fprintf('stop!')
            end
        end
    end
end
Masterout=Master;

% Constants for background enzimes (filters depending on M)
% Autorregulations are CONSIDERED POSITIVE INTERACTIONS.
    function bk=bg(nod,M)
        bkc=0.5; %[Background enzime] for activation.
        bkinc=0.5; %[Background enzime] for inhibition.
        % Fila actua sobre columna. Columna = derivada de cada node.
        bk=zeros(2,nod); % Constant concentration of background
enzimes
        %Row 1= activating enzime.
        %Row 2= inactivating enzime. Each column = each node.
        M(M>1)=-1;%Just in case the matrix has not been traslated
from 2 -> -1
        for i=1:nod
            a=M(:,i); % Recorrer columns. Each column= derivative of
a node.
            if find(a==1) % Does the node have positive incoming
links?
                %Yes, it does = Leaves enzime to 0.
            else
                bk(1,i)=bkc; %No.
            end
            if find(a==-1) % Does the node have negative incoming
links?
                %Yes, it does. Leaves enzime to 0.
            else
                bk(2,i)=bkinc; %No.
            end
            clear a
        end
    end

end
```
