# Supplementary material for: Drug treatment efficiency depends on the initial state of activation in nonlinear pathways
Source: Sci Rep. 2018 Aug 21;8:12495. doi: 10.1038/s41598-018-30913-9 (PMC6104077; doi:10.1038/s41598-018-30913-9)
Supplement: Supplementary file 6 — Matlab scripts [file 41598_2018_30913_MOESM6_ESM.zip › Network_Matlab_scripts/dresfun_main.pdf]

```
%% Dose-response main function:
% input = ind (number) = topology number extracted from
"MasterBC_sel"
% maxit = number of iterations per topology
% output(cell):
%   1st column (matrix)= interaction matrix for each topology.
%   2nd column(struct)= resulting analysis: initial condition
values, response, kinetic
%       parameters, range of concentrations, errors.

% Calls dresfun_it.m
function dresfun_main(ind,maxit)
load('Topologies_raw')

fprintf('Running the topology %i, for %i iterations\n',ind, maxit)
% 2. Globals and seeds for random.
global optiontol reltol tol Lvec inhNode latin_type tspanc timeout
nod h11 h31 IC50
try
    rng('shuffle')
catch
    stream = RandStream('mt19937ar','seed',sum(100*clock));
    RandStream.setDefaultStream(stream)
end

% 3. Parameters:
IC50=1e-7; %IC50 for the inhibitor.
inhNode = 2; % Inhibited node (node B (n=2) is inhibited)
nod =3;
timeout = [500 700 1000]; %time (seconds) spent in each of the 3
steps of the simulation.
tspanc = [1e3 5e3 5e4]; %tvecs (seconds) for each step of the
simulation (before steady state is reached)
h11=1;
h31=1;

% 4. [Inhibitor]:
ligandpoints=25;
L_initial_vectorlog=linspace(log10(1e-4),log10(1e4),ligandpoints);
Lvec=10.^L_initial_vectorlog;

% 5. General Options:
optiontol='yes'; %if no = ode15s selects its own Tolerances.
fig_time=0 ;
latin_type='log'; % Type of constants sampling (logarithmic).
% Ode options:
% toler=1e-5; %abstol not used.
reltol=1e-4; %KEEP reltol LOW TO AVOID yc>1
tol=.05; %values of yc we tolerate upper to 1.
filename = sprintf('Master_ind%d_BCdep',ind);
M_sel=cell(1,2);
M_sel(1,1)=Master(ind,1); %selection inside Master
clear Master
```

```
    Masterout=dresfun_it(M_sel,maxit);
    M_sel=Masterout;
    save(filename,'M_sel')
end
```
