# Supplementary material for: Drug treatment efficiency depends on the initial state of activation in nonlinear pathways
Source: Sci Rep. 2018 Aug 21;8:12495. doi: 10.1038/s41598-018-30913-9 (PMC6104077; doi:10.1038/s41598-018-30913-9)
Supplement: Supplementary file 6 — Matlab scripts [file 41598_2018_30913_MOESM6_ESM.zip › Network_Matlab_scripts/latin.pdf]

```
% Defining Network Topologies that Can Achieve Biochemical
Adaptation:
%
% For each network topology, 10,000 parameter sets were sampled
uniformly in logarithmic
% scale in the np-dimensional parameter space, using the Latin
hypercube sampling method
% (Iman et al., 1980). The sampling ranges of the parameters are
k~0.1-10 and K~0.001-100. A
% circuit refers to a network topology with a particular choice of
parameters.

% Latin hypercube sampling [17] of the initial parameter guesses can
be used
% to guarantee that each parameter estimation run starts in a
different region
% in the high-dimensional parameter space. This method prohibits
that randomly
% selected starting points are accidentally close to each other.
% %% Sampling parameters for a determined range (lb and ub)
% lb = [-10 0]; % lower bound
% ub = [10 1]; % upper bound
% n = 40; % number of samples
% p = 6; % number of parameters
% xn = lhsdesign(n,p); % generate normalized design
% xn=log(xn);
% x = bsxfun(@plus,lb,bsxfun(@times,xn,(ub-lb)));

%% Sampled uniformly in logarithmic scale:

function [kmat,Kmat]=latin(nod,n) %inputs: number of nodes, number
of samples
Kmat=cell(1,3);
kmat=cell(1,3);

kprev=NaN(nod,nod);
Kprev=kprev;

%% A. Matrices for k parameters:
%
lbk=0.1; %lower value for k
ubk=10; %upper value for k
for i=1:n*3
    kprev(:,:,i)=exp(log(lbk) + lhsdesign(nod,nod)*log(ubk/lbk));
end

kmat{1,1}=kprev(:,:,1:i/3); %k constants
kmat{1,2}=kprev(:,:,i/3+1:2*i/3); %kin constants
kmat{1,3}=kprev(:,:,2*i/3+1:end);
%% kmat{1,3} structure (kbk constants + kinput):
% kbk1       kbk2       kbk3
% kbin1      kbin2      kbin3
% kinput     nothing    nothing
```

```
%% B. Matrices for K parameters:
lbK=0.001; %lower value for K
ubK=100; %upper value for K
%In logarithmic scale:
for i=1:n*3
    Kprev(:,:,i)=exp(log(lbK) + lhsdesign(nod,nod)*log(ubK/lbK));
end
Kmat{1,1}=Kprev(:,:,1:i/3); %k constants
Kmat{1,2}=Kprev(:,:,i/3+1:2*i/3); %kin constants
Kmat{1,3}=Kprev(:,:,2*i/3+1:end); %kbk constants + kinput :
end
```
