# Supplementary material for: Drug treatment efficiency depends on the initial state of activation in nonlinear pathways
Source: Sci Rep. 2018 Aug 21;8:12495. doi: 10.1038/s41598-018-30913-9 (PMC6104077; doi:10.1038/s41598-018-30913-9)
Supplement: Supplementary file 6 — Matlab scripts [file 41598_2018_30913_MOESM6_ESM.zip › Network_Matlab_scripts/rcalcfun_fixed.pdf]

```
%  Calls response.m for a number of iterations (maxit); stores the
results.
function [rvec, rvecpos, rvecA, rvecposA,rvecB,rvecposB, high_yc,
sserror,warn,yc0_1,yc0_2,yc0_1expo]= rcalcfun_fixed(yc0)
global Lvec nod

%Inizializing vectors:
rvec=NaN(1,length(Lvec)); % Vector for output node with low inh.
rvecpos=rvec; % Vector for output node with high inh.

rvecA=rvec; % Vector for input node with low inh.
rvecposA=rvec; % Vector for input node with low inh.

rvecB=rvec; % Vector for target node with low inh.
rvecposB=rvec; % Vector for target node with high inh.

yc0_1=nan; %Vector for initial conditions preinitialized.
yc0_1expo=nan;  %Vector for initial conditions with low inh.
yc0_2=nan; %Vector for initial conditions with high inh.

% Counter giving 3 oportunities to the simulation to reach the
steady state
% (controled by timeout):
cvec=1;
% Vecs for errors: 3 cols = [initialization, low inh, high inh]
high_yc=zeros(length(Lvec),3); %if any concentration during the
simulation is >1+tol
warn=high_yc; %if any warn during the simulation.
sserror=high_yc; %if the simulation couldn't reach the steady state.



%% STEADY STATE WHEN L=0;
init=0;
while cvec<4 %cvec= counter for tspanc and timeout;
    [tc,yc,IE,WARN,SSTATE]=response(yc0,0,'15sopt',init, cvec);
%Without drug (L=0);
    if SSTATE==0 && WARN==0 && IE==0 %no errors but sstate=0
        cvec=cvec+1;
        init=max(tc);
    else %no errors, cvec stays the same and data is stored.
        break
    end
end
cvec(cvec>3)=3;

if WARN==1 || IE==1 ||SSTATE==0
    warn(1,1)=WARN;
    high_yc(1,1)=IE;
    sserror(1,1)=1-SSTATE;  %changed to store 1-sstate = sserror.
28/4/2016
    rvec=NaN;
    rvecpos=rvec;
    rvecA=NaN;
```

```
    rvecposA=rvec;
    rvecB=NaN;
    rvecposB=rvec;
else
    yc0_1=yc(end,:);
    j=0;
    %% 1. [LOW INHIBITOR]:
    for i=Lvec
        j=j+1;
        init=0;
        while cvec<4 %cvec= counter for tspanc and timeout;
            [tc,yc,IE,WARN,SSTATE]=response(yc0_1,i,'15sopt',init,
cvec);
            if SSTATE==0 && WARN==0 && IE==0 %no errors but sstate=0
                init=max(tc);
                cvec=cvec+1;
            else %if no errors, cvec stays the same and data is
stored.
                break
            end
        end
        cvec(cvec>3)=3;

        if any(isnan(tc)) || any(any(isnan(yc)))
            rvec=NaN;
            rvecpos=rvec;
            rvecA=NaN;
            rvecposA=rvec;
            rvecB=NaN;
            rvecposB=rvec;
        else
            rvec(j)=yc(end,3);
            rvecA(j)=yc(end,1);
            rvecB(j)=yc(end,2);
        end
        %Expo after the lowest Inh concentration is applied.
        if i==Lvec(1) && any(any(isnan(yc)))==0 %yc0_1expo is stored
if yc worked.
            yc0_1expo=yc(end,:);
        end

        if WARN==1 || IE==1 ||SSTATE==0
            warn(j,2)=WARN;
            high_yc(j,2)=IE;
            sserror(j,2)=1-SSTATE;  %changed to store 1-sstate =
sserror. 28/4/2016
            rvec=NaN;
            rvecpos=rvec;
            rvecA=NaN;
            rvecposA=rvec;
            rvecB=NaN;
            rvecposB=rvec;
            break
        else
```

```
        end
    end
    if isnan(rvec) %if 15s doesn't work either, rvec and rvecpos =
NaN
        % and func ends HERE.
    else % rvec has been created successfully.
      %% 2. [HIGH INHIBITOR]:
        j=0;
        yc0_2=yc(end,:);
        yc0_2(yc0_2>1)=1; %rounding negative close-to-zero values to
zero:
        %cvec is cumulative of all the function.
        for i=fliplr(Lvec)
            j=j+1;
            init=0;
            while cvec<4 %cvec= counter for tspanc and timeout;

[tc2,yc2,IE,WARN,SSTATE]=response(yc0_2,i,'15sopt',init,cvec);
                if SSTATE==0 && WARN==0 && IE==0 %no errors but
sstate=0
                    init=max(tc2);
                    cvec=cvec+1;
                else % sstate reached or errors not solved with
higher times.
                    break
                end
            end
            cvec(cvec>3)=3;

            if any(isnan(tc2)) || any(any(isnan(yc2)))
                rvec=NaN;
                rvecpos=rvec;
                rvecA=NaN;
                rvecposA=rvec;
                rvecB=rvec;
                rvecposB=rvec;

            else
                rvecpos(j)=yc2(end,3);
                rvecposA(j)=yc2(end,1);
                rvecposB(j)=yc2(end,2);
            end
            if WARN==1 || IE==1 ||SSTATE==0
                warn(j,3)=WARN;
                high_yc(j,3)=IE;
                sserror(j,3)=1-SSTATE;
                rvec=NaN;
                rvecA=NaN;
                rvecpos=rvec;
                rvecposA=rvec;
                rvecB=NaN;
                rvecposB=NaN;
                break
            else
```

```
            end
        end
        if isnan(rvec)
        else % rvecpos has been created succesfully.
        end
    end
end
end
```
