# Supplementary material for: Drug treatment efficiency depends on the initial state of activation in nonlinear pathways
Source: Sci Rep. 2018 Aug 21;8:12495. doi: 10.1038/s41598-018-30913-9 (PMC6104077; doi:10.1038/s41598-018-30913-9)
Supplement: Supplementary file 6 — Matlab scripts [file 41598_2018_30913_MOESM6_ESM.zip › Network_Matlab_scripts/response.pdf]

```
function
[tc,yc,IError,WARN,SSTATE]=response(yc0,L,solver,init,counter)
global kprev kinprev K Kin kbk Kbk bk tspanc toler reltol nod
inhNode M cstim stim timeout tol h11 h31 IC50%fig_time
lastwarn('')
errvec=[1 2 3];
prev=50; npoints=5; thres=5e-4;
IE = [];
sstate = 0;
I=L; % [Inhibitor]
k=kprev;
kin=kinprev;

%% Inhibitor effect in target node:
for i=1:nod;
    k1=1; k_1=IC50;ktot=kprev(inhNode,i); kintot=kinprev(inhNode,i);
    k(inhNode,i) = ktot*k_1/(k1*I + k_1);
    kin(inhNode,i) = kintot*k_1/(k1*I + k_1);
end
TimeOut=timeout(counter); xoverFcn = @(T, Y) MyEventFunction(T, Y,
TimeOut,tol);
options=odeset('RelTol', reltol,'AbsTol',toler,'Events',xoverFcn);

tic;
try
    [tc1,yc1,TE,YE,IE] = ode15s(@allnodes_stim,[init
tspanc(counter)],yc0,options);
    error(lastwarn)
catch
    tc1=NaN;
    yc1=NaN;
    lastwarn('')
end

if isnan(yc1) % Warning error appear. DO NOT CONTINUE.
    yc=yc1;
    tc=tc1;
else
    sstate=sstate_test(tc1,yc1,prev,npoints,thres);

    if ~isempty(IE) && any(ismember(errvec,IE))
        yc=NaN;
        tc=NaN;
    elseif sstate==1 % Reached S STATE || yc> 1+tol (IE Event
flagged)
        yc=yc1; tc=tc1;
    else %sstate==0 Steady state not reached.
        yc=yc1; tc=tc1;
    end
end
%% Error tracking:

if isempty(IE) || sum(IE)==0 || IE(end)==nod+1%IE=3 or 4 means
timeout = 1.
```

```
    IError=0;
else
    IError=1; %values higher than yc + tol
end

SSTATE=sstate;

if isnan(yc)
    WARN=1;
else
    WARN = 0;
end

clear k kin
%% Functions:
    function dydt = allnodes_stim(t,x,TimeOut) %december 2015
        % (1) CONSTANT activation of node A by adding a constant
stimuli (stim).
        % (2) INHIBITION by making kcat's coming from the inhibited
edges PROPORTIONAL to [inhibitor], following a Hill function.
        % Inhibition prevents the action (phophorilation) of the
node (and that's why we are changing the kcat's).
        % (3) No +bk enzime for node A: it already receives a
constant stimuli
        % (stim).

        % k and kin filtering depending on M:
        k(M<=0)=0; %values with 0/-1 = no pos interaction
        kin(M>=0)=0; %values with 0/1 = no neg interaction
        dydt=   [x(1)*k(1,1)*((1-x(1))^h11)/(((1-x(1))^h11)+
(K(1,1))^h11) + x(2)*k(2,1)*(1-x(1))/((1-x(1))+K(2,1)) +
x(3)*k(3,1)*(1-x(1))/((1-x(1))+K(3,1)) + (stim-x(1))*cstim + ...
            - x(1)*kin(1,1)*x(1)/(x(1)+Kin(1,1))     -
x(2)*kin(2,1)*x(1)/(x(1)+Kin(2,1))     - x(3)*kin(3,1)*(x(1)^h31)/
((x(1)^h31)+(Kin(3,1)^h31))     - bk(2,1)*kbk(2,1)*x(1)/
(x(1)+Kbk(2,1));    %x(1) = 'A'

            x(1)*k(1,2)*(1-x(2))/((1-x(2))+K(1,2)) + x(2)*k(2,2)*(1-
x(2))/((1-x(2))+K(2,2)) + x(3)*k(3,2)*(1-x(2))/((1-x(2))+K(3,2)) +
bk(1,2)*kbk(1,2)*(1-x(2))/((1-x(2))+Kbk(1,2)) ...
            - x(1)*kin(1,2)*x(2)/(x(2)+Kin(1,2))     -
x(2)*kin(2,2)*x(2)/(x(2)+Kin(2,2))     - x(3)*kin(3,2)*x(2)/
(x(2)+Kin(3,2))     - bk(2,2)*kbk(2,2)*x(2)/(x(2)+Kbk(2,2));

            x(1)*k(1,3)*(1-x(3))/((1-x(3))+K(1,3)) + x(2)*k(2,3)*(1-
x(3))/((1-x(3))+K(2,3)) + x(3)*k(3,3)*(1-x(3))/((1-x(3))+K(3,3)) +
bk(1,3)*kbk(1,3)*(1-x(3))/((1-x(3))+Kbk(1,3)) ...
            - x(1)*kin(1,3)*x(3)/(x(3)+Kin(1,3))     -
x(2)*kin(2,3)*x(3)/(x(3)+Kin(2,3))     - x(3)*kin(3,3)*x(3)/
(x(3)+Kin(3,3))     - bk(2,3)*kbk(2,3)*x(3)/(x(3)+Kbk(2,3))];
    end

    function [VALUE, ISTERMINAL, DIRECTION] =
MyEventFunction(t,y,TimeOut,tol)
```

```
        %The event function stops the intergration is VALUE == 0 and
        %ISTERMINAL==1
        %         dydt = allnodes_stim(t,y,z);
        %         sstate = norm(dydt) - 1e-5;
        %or -1, doesn't matter
        %         if t>99
        %         end
        %%a. Define the timeout in seconds CHANGED FOR dde23
1/2/2016
        %%b. The solver runs until this VALUE is negative (does not
change the sign)
        %         tol=0.5;
        VALUE=[y(1)-(1+tol);y(2)-(1+tol);y(3)-(1+tol);(TimeOut-
toc)];
        ISTERMINAL = [1;1;1;1];
        DIRECTION = [0;0;0;0]; %d. The direction does not matter
    end
    function sstate=sstate_test(tc,yc,prev,npoints,thres)
        if max(tc)<prev; % max(tc) needs to be at least 50.
            ycvec=nan;
        else
            ycend=find(tc>(max(tc)-prev));
            if length(tc)<6 %we cannot take the last 5 points if tc
is >=5.
                ycvec=nan;
            elseif length(ycend)<100 %if the 5% of the curve has
less than 100 points:
                ycvec=yc(end-5:end,:); %From the last 100 points we
take 5.
            else

ycend_points=round(linspace(min(ycend),max(ycend),npoints));
                ycvec=yc(ycend_points,:);
            end
        end
        if any(any(isnan(ycvec))) ||
any(any(abs(diff(ycvec))>thres))
            sstate=0;%S.STATE NOT REACHED =>continue (cvec counts
the tries)
        else
            sstate=1;%S.STATE REACHED.
        end
    end

end
```
